# Retraction of P-1395. Comparative Analysis of Sensitivity and Add-On Value of Xpert and Xpert Ultra in Diagnosing Pulmonary Tuberculosis and Stool Samples of Children in Indian

**DOI:** 10.1093/ofid/ofag308

**Published:** 2026-06-11

This is a retraction of Monirujjaman Biswas, P-1395. Comparative Analysis of Sensitivity and Add-On Value of Xpert and Xpert Ultra in Diagnosing Pulmonary Tuberculosis and Stool Samples of Children in Indian, *Open Forum Infectious Diseases*, Volume 13, Issue Supplement_1, January 2026, ofaf695.1582, https://doi.org/10.1093/ofid/ofaf695.1582.

The journal is retracting this IDWeek abstract because there is significant overlap between this abstract and a previously published abstract without attribution: Evaluation of Xpert MTB/RIF Assay on Stool Samples for the Diagnosis of Pulmonary Tuberculosis among the Pediatric Population (10.1055/s-0042-1757721). Additionally, the authorship of the publication cannot be verified.

